# A survey study of healthcare workers on do not Attempt cardiopulmonary resuscitation practice and policy in Ireland

**DOI:** 10.1016/j.resplu.2024.100799

**Published:** 2024-10-17

**Authors:** John Lombard, Hope Davidson, Owen Doody

**Affiliations:** a School of Law, University of Limerick, Limerick, Ireland; b Health Research Institute, Department of Nursing and Midwifery, University of Limerick, Limerick, Ireland

**Keywords:** Do not attempt cardiopulmonary resuscitation, End-of-life, Decision-making, Healthcare professional, Policy

## Abstract

**Aim:**

Do Not Attempt Cardiopulmonary Resuscitation (DNACPR) codes record the decision to withhold CPR in cases of circulatory arrest. These decisions involve various clinical, ethical and legal complexities promoting increased focus on the decision-making process. This research sought to capture healthcare workers perspective on DNACPR practices and policies in Ireland.

**Methods:**

A cross-sectional descriptive survey utilising a questionnaire developed and piloted for this study to gather responses on open and closed questions. Data were analysed using SPSS and content analysis. Study is reported in line with the Consensus-Based Checklist for Reporting of Survey Studies reporting guidelines.

**Results:**

784 participants including doctors, nurses, paramedics and other healthcare workers completed the questionnaire. 80.5 % (n = 625) of participants rated their knowledge of DNACPR decision-making as fair or better. 77.5 % (n = 601) of participants understood DNACPR to mean ‘no chest compressions, defibrillation or artificial ventilation in the event of cardiopulmonary arrest’. A majority of participants (60.2 % n = 467) had experienced a degree of conflict related to a DNACPR decision. 245 (31.25%) participants provided comments which addressed issues such as communication, education, pressure surrounding DNACPR decisions, the role of national guidelines/documentation, and legal concerns.

**Conclusion:**

The findings reveal gaps in healthcare workers' understanding and familiarity with DNACPR policies, highlighting the need for improved patient involvement and proactive discussions. Effective communication and comprehensive training are crucial, as communication remains a significant barrier. While national policies can provide clarity, increasing awareness and understanding of these policies among healthcare workers is essential.

## Highlights

• Deficiencies identified in understanding of DNACPR among healthcare professionals.
• A majority of participants experienced conflict related to a DNACPR decision.
• Communication difficulties are a significant barrier to good decision-making.
• Greater awareness of national DNACPR policy is required.

## Introduction

A decision to withhold cardiopulmonary resuscitation (CPR) may be documented in advance using an appropriate Do Not Attempt Cardiopulmonary Resuscitation (DNACPR) form. These decisions involve complex clinical, ethical, and legal considerations. In Ireland, the COVID-19 pandemic highlighted the complexity of DNACPR decisions and emphasised the crucial role of healthcare workers in this decision-making process. The principal guidance for DNACPR in Ireland is provided by the Health Service Executive (HSE) National Consent Policy^1^ which was supplemented during the pandemic by the ‘HSE Guidance Regarding Cardiopulmonary Resuscitation and DNAR Decision-Making during the COVID-19 Pandemic'.^2^ The Irish healthcare system comprises both public and private sectors with the HSE serving as the main public body responsible for providing health and social care services. The HSE guidance applies to all providers offering services on behalf of the HSE, including the ambulance service, acute and community hospitals, and long-stay care settings.

In terms of the legal framework, Irish law recognises an individual's right to refuse medical treatment, even if such a refusal results in death. This means a person can opt to refuse CPR and request that a DNACPR decision be documented. When a person lacks the capacity to make such a decision, resuscitation decisions are based on the person’s best interests, which include not only clinical factors but also the individual’s known values and preferences.

While the HSE’s guidance on DNACPR is shaped by legal and ethical principles, many healthcare institutions develop local policies tailored to their specific circumstances. Furthermore, there is currently no national standardised DNACPR form, leading to variability in documentation of decisions across healthcare providers. Previous research in Ireland has highlighted gaps in the understanding of DNACPR decisions.^3^, ^4^, ^5^ However, these studies were often limited to a small number of hospital settings, had relatively small sample sizes, and some were conducted before the publication of the COVID-19 DNACPR guidance. This article outlines empirical research aimed at capturing and describing the perspectives and understanding of healthcare workers in Ireland regarding DNACPR practice and policy. Specifically, this paper explores DNACPR knowledge, decision-making, communication and adherence to DNACPR decisions. This research was undertaken to inform the development of a single national policy for DNACPR decision-making in Ireland, encompassing CPR and DNACPR decisions within the broader framework of consent.

## Methods

A questionnaire was developed through a literature review,^6^ the analysis of DNACPR-related complaints and incidents recorded in sources such as the National Incident Management System, and a review of international practices and policies in jurisdictions such as England, Wales, Ohio (United States), Australia, New Zealand, and British Columbia (Canada).^7^ Stakeholder engagement also informed the development process. The HSE DNACPR project working group, consisting of legal and medical professionals as well as patient representatives, served as the primary stakeholder group, providing feedback at each stage of questionnaire’s development. Additional feedback was gathered from three healthcare workers and four colleagues experienced in quantitative research. Feedback focused on face and content validity, leading to minor edits, which were agreed upon by all contributors. The study adhered to the Consensus-Based Checklist for Reporting of Survey Studies guidelines (Supplementary File S1).^8^.

The final questionnaire distributed via Qualtrics comprised of 40 questions organised into six sub-sections: demographic and career characteristics (questions 1–9), DNACPR knowledge and policy (questions 10–17), timing of DNACPR decision-making (questions 18–21), involvement in DNACPR decision-making (questions 22–30), DNACPR discussion (questions 31–34), and communication of and adherence to the DNACPR decision (questions 35–39). The final question (question 40) was open-ended, allowing participants to provide additional comments. A range of question types was used including close-ended questions, matrix questions, Likert scales and filtering questions.

The questionnaire and accompanying information letter were circulated to healthcare workers via an HSE all-staff communication, HSE social media channels, and through relevant stakeholders such as the Irish Medical Council. Responses were collected anonymously between 11 October 2023 and 24 November 2023. In total, 784 participants completed the questionnaire. Data were analysed using SPSS and Cronbach’s alpha was calculated to assess internal consistency or reliability, yielding a good reliability score (α.79). A power analysis, based on HSE employment data, indicated that the required sample size for a population of 120,000 staff members, with a 90 % confidence level and a 5 % margin of error, was approximately 270 respondents. Missing data was excluded from statistical analysis. Qualitative data from open-ended questions were analysed utilising Elo and Kyngas’s content analysis framework.^9^.

Ethical approval for the study was granted by the Health Service Executive Research Ethics Committee (ref: 055/2023). The questionnaire landing page required participants to provide consent before beginning. Participants were assured of their right to skip questions, withdraw at any time, and that all responses would remain anonymous.

## Results

Of the 784 participants, the majority were women (64 %), with 35 % men. All age groups were represented, and participants included doctors, nurses and paramedics and full demographics are presented in Table 1. Of the 19.8 % who selected ‘other’ for their profession included roles such as emergency medical technician, advanced paramedic, physiotherapist, and healthcare assistant.

**Table 1:** Participant demographic profile.

**Demographic detail**	Descriptor	% n= (response)
	Man	35 % n = 271
	Woman	64 % n = 498
**Gender**	Non-binary	0 % n = 0
	I identify my gender as:	1 % n = 6 (male n = 4, female n = 2)
	Prefer not to say	1 % n = 5
	18–29 years	6.3 % n = 49
	30–39 years	21.1 % n = 165
	40–49 years	33.1 % n = 259
**Age**	50–59 years	30.3 % n = 237
	60–69 years	8.7 % n = 68
	70–79 years	0.5 % n = 4
	80 + years	0.1 % n = 1
	Doctor	13 % n = 101
**Profession**	Nurse	42.4 % n = 330
	Paramedic	24.8 % n = 193
	Other	19.8 % n = 154
	Intern	0.6 % n = 5
	Senior House Officer	1.4 % n = 11
	Registrar	1 % n = 8
	Specialist Registrar	1.8 % n = 14
	Consultant	7.7 % n = 60
	General Practitioner	0.8 % n = 6
	Staff nurse	5.2 % n = 40
**Job title**	Clinical Nurse Manager	17.3 % n = 134
	Clinical Nurse Specialist	5.3 % n = 41
	Advanced Nurse Practitioner	3.1 % n = 24
	Emergency Medical Technician	6.6 % n = 51
	Paramedic	12.9 % n = 100
	Advanced Paramedic	10.6 % n = 82
	Other	23.6 % n = 183
	Not applicable	2.1 % n = 16
**Years experience**	Less than one year	1.5 % n = 12
	1 – 5 years	14 % n = 109
	6 – 10 years	14.2 % n = 111
	11 – 15 years	14.9 % n = 116
	16 + years	55.3 % n = 431
**Qualification obtained**	Ireland	79.1 % n = 613
	United Kingdom	12.3 % n = 95
	India	2.5 % n = 19
	United States	0.6 % n = 6
	Philippines	0.6 % n = 5
	Pakistan	0.6 % n = 5
	Other countries	4.3 % n = 33
**Involved in a DNACPR decision-making process as a healthcare worker**	In the previous twelve months	50.3 % n = 393
	Not involve (last 12 months)	49 % n = 383
	Unsure	0.8 % n = 6

### DNACPR knowledge and policy

Participants rated their familiarity with a range of DNACPR-related policies, legislation, and decision-making instruments (Table 2). Sub-scale reliability achieved α.855. Of the participants, 26.5 % (n = 205) were very or extremely familiar with the ‘DNAR’ guidance contained in the National Consent Policy.

**Table 2:** Familiarity with policy, legislation, and decision-making instruments.

	**Not at all familiar**	**Slightly familiar**	**Moderately familiar**	**Very familiar**	**Extremely familiar**
HSE National Consent Policy 2022	9 % n = 70	15.5 % n = 119	37.2 % n = 288	30.1 % n = 233	8.3 % n = 64
Guidance on ‘Do Not Attempt Resuscitation’ contained in the HSE National Consent Policy 2022	18.9 % n = 146	22.1 % n = 171	32.5 % n = 251	20.8 % n = 161	5.7 % n = 44
HSE Guidance Regarding Cardiopulmonary Resuscitation and DNAR Decision-Making during the COVID-19 pandemic	20.50 % n = 158	19.2 % n = 148	29.4 % n = 226	22.3 % n = 172	8.6 % n = 66
The Assisted Decision-Making (Capacity) Act 2015	7.2 % n = 55	15.9 % n = 122	32.8 % n = 252	32.7 % n = 251	11.5 % n = 88
Advance Healthcare Directives	9.4 % n = 72	19.1 % n = 146	33.2 % n = 254	30.4 % n = 233	8 % n = 61
Decision support arrangements under the Assisted Decision-Making (Capacity) Act (e.g., decision-making assistant; co-decision-maker; decision-making representative; designated healthcare representative)	13.5 % n = 104	21.6 % n = 166	32.7 % n = 251	24.9 % n = 191	7.3 % n = 56

Several questions assessed participants’ knowledge of DNACPR and CPR. 80.5 % (n = 625) rated their knowledge of DNACPR decision-making as fair or better. Additionally, 77.5 % (n = 601) understood DNACPR to mean ‘no chest compressions, defibrillation or artificial ventilation in the event of cardiopulmonary arrest’, while 14.5 % (n = 112) understood it as a ‘limitation of measures to preserve life which extend beyond resuscitation’, and 8 % (n = 62) believed it to mean ‘no effort should be made to preserve life’. In relation to CPR survival rates, 25.2 % (n = 196) of participants estimated the survival rate to hospital discharge after in-hospital cardiac arrest to be 35–49 % or higher. Conversely, 70.1 % (n = 545) considered the survival rate for an out-of-hospital cardiac arrest to be less than 10 %.

Participants rated the importance of various factors in DNACPR decision-making using a matrix table (Table 3). Sub-scale reliability achieved α.761.

**Table 3:** Elements informing DNACPR decision-making.

	**Not at all important**	**Slightly important**	**Moderately important**	**Very important**	**Extremely important**
National policy, e.g., HSE National Consent Policy 2022	1.3 % n = 10	4.9 % n = 38	15.8 % n = 121	41.4 % n = 318	36.6 % n = 281
Local policy	5.1 % n = 39	6.3 % n = 48	22.5 % n = 172	38.3 % n = 293	27.8 % n = 213
Patient wishes and preferences	0.1 % n = 1	0.4 % n = 3	2.6 % n = 20	15 % n = 115	81.9 % n = 630
Advance healthcare directive	0.3 % n = 2	1.3 % n = 10	9.3 % n = 71	31.1 % n = 238	58.1 % n = 445
Patient's quality of life	0.70 % n = 5	0.80 % n = 6	3 % n = 23	22 % n = 169	73.5 % n = 564
Input of patient's family members	2 % n = 15	12 % n = 92	32.3 % n = 247	32.8 % n = 251	20.9 % n = 160
Input of decision supporters under the Assisted Decision-Making (Capacity) Act (e.g., decision-making assistant; co-decision-maker; decision-making representative; designated healthcare representative)	0.3 % n = 2	3.9 % n = 30	19 % n = 145	43.2 % n = 330	33.6 % n = 257
Professional standards / code of conduct	0.4 % n = 3	2 % n = 15	6.8 % n = 52	34 % n = 261	56.8 % n = 436
Personal clinical judgement	2.6 % n = 20	6.9 % n = 53	19.7 % n = 152	38.2 % n = 295	32.7 % n = 253
Input from other healthcare workers	1.8 % n = 14	6.9 % n = 53	24.8 % n = 192	40.1 % n = 310	26.4 % n = 204

### Timing of DNACPR Decision-Making

Participants identified the most appropriate time to introduce discussions about DNACPR with participants able to select multiple options. 434 participants selected ‘while attending the GP surgery’, 529 selected ‘after a person is diagnosed with a terminal illness’, 219 selected ‘during an outpatient clinic appointment’, 378 selected ‘after a person is admitted to hospital’, 282 selected ‘at the time of consent for surgery’. Additionally, 32 participants were ‘not sure’, and 174 participants proposed another time.

Regarding time spent discussing resuscitation status with patients, 32 % (n = 131) typically spend over 15 min, while 11.7 % (n = 48) spend less than one minute, 22.2 % (n = 91) spend 1–5 or 6–10 min, and 12 % (n = 49) spend 11–15 min.

Participants were also asked how likely it would be to reassess a documented DNACPR decision in nine specific scenarios (Table 4). Sub-scale reliability achieved α.784.

**Table 4:** Review of DNACPR decision.

	**Extremely unlikely**	**Somewhat unlikely**	**Neither likely nor unlikely**	**Somewhat likely**	**Extremely likely**
**The patient’s clinical condition deteriorates**	15.6 % n = 119	12.6 % n = 96	9.8 % n = 75	31.7 % n = 242	30.4 % n = 232
**The patient’s clinical condition improves**	5.8 % n = 44	15.9 % n = 121	16 % n = 122	39.2 % n = 299	23.2 % n = 177
**The patient’s preferences regarding CPR change**	1.3 % n = 10	2.8 % n = 21	7 % n = 53	24.8 % n = 188	64.2 % n = 487
**A patient has recovered capacity in respect of a DNACPR decision**	1.8 % n = 14	5 % n = 38	7.4 % n = 56	29.6 % n = 225	56.2 % n = 428
**An advance healthcare directive is identified**	2.4 % n = 18	3.7 % n = 28	9.9 % n = 75	34.3 % n = 260	49.7 % n = 376
**Clinical responsibility for the patient changes**	4.7 % n = 35	10.1 % n = 76	19.2 % n = 144	38.6 % n = 290	27.4 % n = 206
**The patient is to undergo a medical or surgical procedure**	5.3 % n = 40	8.6 % n = 65	16.8 % n = 127	38.6 % n = 291	30.6 % n = 231
**The patient has a prolonged stay in hospital or is a nursing home resident**	8.5 % n = 65	12.2 % n = 93	16.4 % n = 125	35.6 % n = 271	27.2 % n = 207
**At time of hospital discharge**	17.9 % n = 136	21.6 % n = 164	23.4 % n = 178	24.4 % n = 186	12.7 % n = 97

The final question in this section asked whether a DNACPR decision from a prior hospital admission could be used if the patient is readmitted. In response, 38.1 % (n = 295) answered ‘yes’, 31.7 % (n = 246) ‘no’, and 30.2 % (n = 234) were ‘not sure’.

### Involvement in DNACPR Decision-Making

Regarding patient involvement in DNACPR decision-making, 75.7 % (n = 585) believed that a patient with the requisite capacity is often or always involved. Additionally, 64.9 % (n = 502) thought the patient is often or always given the opportunity to involve a family member, friend, or decision supporter. However, when CPR is deemed inappropriate, 48.3 % (n = 372) believed this decision is often or always communicated to the patient, while 11.8 % (n = 78) thought it is never or rarely communicated, 18.9 % (n = 146) said it is only communicated ‘sometimes’, and 21 % (n = 162) were ‘not sure’.

66.8 % (n = 519) strongly agreed that a person’s refusal to participate in DNACPR decision-making should be respected. When asked about the statement: ‘a patient does not need to be included in the DNACPR decision-making process if this would be upsetting for them.’ 52.4 % (n = 407) strongly disagreed, 22.3 % (n = 173) somewhat disagreed, 6.8 % (n = 53) neither agreed nor disagreed, 10.4 % (n = 81) somewhat agreed, 5.9 % (n = 46) strongly agreed, and 2.1 % (n = 16) were not sure.

60.2 % (n = 467) experienced tensions regarding a DNACPR decision with a patient, family member, or decision supporter, while 8.9 % (n = 69) were not sure, and 30.9 % (n = 240) had not experienced such tension. Among the 372 participants who provided further detail five themes emerged: demands for CPR, internal family disagreement, lack of understanding, poor communication, and legal uncertainty.

### DNACPR discussion

This section asked participants how comfortable they felt discussing DNACPR with a patient. Of the responses, 21.3 % (n = 136) reported feeling somewhat or very uncomfortable, 12.3 % (n = 79) were neither comfortable nor uncomfortable, and 66.5 % (n = 425) felt somewhat or very comfortable. Additionally, 58.7 % (n = 452) rated their communication skills for DNACPR discussions as good or excellent, while 4.5 % (n = 35) rated them as very poor or poor, 19.7 % (n = 152) as fair, and 17 % (n = 131) were not sure. Participants were also asked who should initiate DNACPR discussions, with multiple selections allowed. The majority, (n = 624), selected a senior healthcare professional, followed by a General Practitioner (n = 546), the patient (n = 513), and a nurse (n = 402). 33 participants were not sure who should initiate the discussion, while 156 suggested others such as paramedics, family members, chaplain, and healthcare assistant. Several comments indicated that all parties involved in the patient’s care should have the responsibility to initiate the discussion.

### Communication of and adherence to the DNACPR decision

In the final sub-section participants were asked about the communication and adherence to DNACPR decisions. Of the respondents 49 % (n = 380) believed the DNACPR decision was always communicated to relevant healthcare workers, while 40.3 % (n = 313) felt this communication did not always occur, and 10.7 % (n = 83) were not sure.

Participants were asked if a second opinion should be offered when there is disagreement about the risks and benefits of CPR. A strong majority 85.1 % (n = 655) agreed that a second opinion should be offered, 2.5 % (n = 19) disagreed, and 12.5 % (n = 96) were not sure.

The penultimate question asked if a DNACPR decision should be respected when a person experiences a cardiorespiratory arrest from a readily reversible cause unrelated to their underlying condition. In response, 39.9 % (n = 308) were not sure, 28.1 % (n = 217) believed the original decision should be followed, and 32 % (n = 247) thought the DNACPR decision should not be respected.

### Open-text responses

A total of 245 participants provided additional comments, primarily addressing issues such as communication, education, pressure surrounding DNACPR decisions, the role of national guidelines and documentation, and legal concerns.

Communication (n = 70).

Communication was the predominant theme, with participants emphasising the importance of DNACPR discussions, the need for communication skills training, and ensuring that all parties are well-informed. Some comments highlighted that DNACPR conversations are sometimes not held with patients, leading to paternalistic decision-making. Participants noted that effective communication might require multiple discussions and involvement from family members, friends, or decision supporters.

*‘Communication in a full and understanding way should be provided to the patient at all times. This may mean a number of discussions with the patient to ensure the patient has full understanding. My experience is that the DNACPR conversation happens once usually with no family involvement and when the patient is in a vulnerable state.’*

Both individuals and their family members should be well-informed about cardiopulmonary resuscitation.

*‘DNACPRs should be discussed more openly with patients and families. Families often don’t understand the risks of CPR and what the likely outcome is to be for their family members.’*

Several participants called for DNACPR communication training for staff.

*‘All staff of all grades should be involved in end-of-life discussions including DNACPR. All staff grades should practice the conversation through role play/scenario education. Senior staff especially medical staff should guide and support junior staff in how to have the discussion and demonstrate good evidence-based practice in communication during difficult conversations – either in the community or the acute setting.’*

Education (n = 43).

Participants emphasised the need for comprehensive education and training on all aspects of DNACPR, including when to have DNACPR discussions, issues surrounding consent, the Assisted Decision-Making (Capacity) Act 2015, and public education about CPR.

*‘Further education for healthcare providers that DNACPR does not mean do not act to reverse infection/dehydration other clearly reversible factors.’*

*‘There is a need for education for medical and nursing staff to improve confidence in following advance directives.’*

*‘General population is not aware how traumatic CPR is, we all need education and training in communicating in lay man terms what CPR involves and the likely outcome.’*

Pressure surrounding the DNACPR decision (n = 24).

Some participants noted the challenges faced by family members or healthcare professionals due to pressure surrounding DNACPR decisions, especially when decisions were delayed or missed.

*‘Families should not be the overall decision makers. Where their involvement is essential medical teams place too much of the decision on families. The burden of this is unfair. How the information is shared with family is so important. Clarity on who is and who can make the decision: Patient, Doctor, Family. I have spoken with family years after the death of a loved one and they still carried the guilt of thinking they made the decision to let their loved one die.’*

*‘Earlier discussion is vital on this subject particularly, far too often left until the patient deteriorates…generally at 03.00 in the morning with no willing decision makers.’*

The role of national guidelines and documentation (n = 50).

Many participants called for clearer national guidance on DNACPR decisions and the introduction of a standardised national form to replace locally developed documents.

*‘There is a need for a standardised DNACPR document, this would avoid the need to repeat distressing conversations for the individual and/or their families. It would also ensure their wishes are respected.’*

*‘National consensus on the management of DNACPR policies and practices needs to be found, as it is too often left to individual institutions or medical teams to come up with their own interpretation of existing practices.’*

Legal concerns (n = 20).

Concern about the legal implications of DNACPR decisions were raised, particularly by pre-hospital healthcare workers. Participants expressed uncertainty about the validity and applicability of DNACPR decisions.

*‘For pre-hospital care these are a grey area that needs much more guidance, we follow basic guidance from PHECC (Pre-Hospital Emergency Care Council) … It’s a difficult area to be in from a prehospital perspective, the law seems to be grey around DNRs. Myself and colleagues in the NAS (National Ambulance Service) feel very wary about these and approach them with little confidence, most are signed two years previous, and most care staff fail to alert us to them during handover.’*

## Discussion

The results reveal significant misunderstandings amongst healthcare staff regarding the scope and application of a DNACPR decisions. These include uncertainties about the legal context, complexities in implementation such as communication with patients and families, and challenges in conveying decisions to clinical staff often leading to tensions with family members. Healthcare professionals would benefit from enhanced training and structured resources to support effective implementation of DNACPR decisions.

A decision to attempt CPR is not purely clinical and often requires patient input. It is vital that healthcare workers provide accurate information and guidance to facilitate informed decision-making.^10^ It is therefore of concern that the results demonstrated gaps in understanding both in the effectiveness of CPR and implications of a DNACPR decision. For instance, 22.5 % (n = 174) of participants believed DNACPR decisions were more restrictive than they actually are. Similarly, in a previous Irish study, 35 % of hospital doctors mistakenly thought DNACPR orders applied to treatments beyond resuscitation.^5^ In the current study, over 25 % of participants overestimated CPR’s effectiveness, with 25.2 % (n = 196) believing the rate of survival for in-hospital cardiac arrest was between 35 % and 49 %, while actual survival rates range from 15 % to 34 %^11^, ^12^, ^13^, ^14^. Estimates for out-of-hospital cardiac arrest survival were similarly inflated, with 30 % (n = 233) of participants estimated survival rates higher than 10 %, whereas the true figure is closer to 8.8 %.^15^ These misjudgements may reflect participants’ personal experiences in specific care contexts. Despite 80.5 % (n = 625) of participants rating their DNACPR knowledge as fair or better, some remain unaware of the gaps in their understanding.

Involving patients in DNACPR decisions is crucial, as it respects their human rights^16^ and can improve patient care.^17^ Patient wishes are considered to be a priority^18^ and this is reflected in the results of this study where 96.9 % (n = 745) of participants emphasised the importance of considering patient wishes. Furthermore, 75.7 % (n = 585) of participants reported that patients with the capacity to contribute were often or always involved in DNACPR decision-making. This compares favourably with other studies where patient involvement ranged from 33 % to 55.8 %.^19^, ^20^, ^21^, ^22^ However, the quality of this involvement warrants scrutiny. For instance, MacCormick noted that doctors hold varying views on what meaningful patient involvement entails.^23^ General Practitioners also differ significantly in how they engage patients and families in decision-making^24^ as reflected in the variability of time participants spent discussing resuscitation status with patients. The results suggest patient involvement often diminishes after a decision is made. For instance, in circumstances where CPR is judged inappropriate less than half (48.3 % n = 372) of participants believed that patients are frequently informed when CPR is deemed inappropriate. This may be due to barriers like cognitive or mental limitations at that time.^25^.

Participants recognised that DNACPR discussions can occur at various stages, not just in the late stages of illness. Early discussions, as previously recommended,^26^, ^19^, ^27^, ^28^ allow patients to fully engage in DNACPR decisions^29^ and involve familiar healthcare workers.^17^ Responsibility for initiating this discussion appeared diffuse, with participants suggesting that it should be shared among all parties. A study by Saltbæk et al demonstrated the ambiguity in documented DNACPR conversations, regarding who initiated the discussion and how decision-making power was distributed.^30^ Although DNACPR discussions may arise organically, having a clear line of responsibility is essential.

A majority of participants felt comfortable discussing DNACPR and rated their communication skills highly. Nonetheless, poor communication emerged as key source of conflict, frequently cited in open-ended responses. Training in DNACPR communication is a recurring theme in literature, with previous studies identifying communication difficulties as a major barrier to DNACPR discussions.^18^ Sterie et al emphasised the need for training on patient involvement in care related discussions.^31^ While Becker et al recommended workshops focusing on patients’ values and goals of care,^32^ Aljohaney and Bawazir^33^ advocated for evidence-based curriculum to improve DNACPR discussions and Chen et al,^34^ Einstein et al.,^27^ El Sayed et al.,^35^ and Fan and Hsieh^36^ highlight the need for training to enhance DNACPR communication skills.

The importance of training and education also extends to familiarity of existing policies. National policies can clarify the decision-making framework for healthcare workers, patients and family members. However, greater awareness and understanding of these policies is a necessary first step as just 26.5 % (n = 205) of participants were very or extremely familiar with the guidance set out in the National Consent Policy.

In considering these findings, it is necessary to acknowledge the limitations of this including the possibility of participants providing socially desirable rather than truthful responses. Additionally, the data represents a single point in time, making it difficult to assess changes over time, and the survey’s depth is limited in exploring participants' experiences and perspectives. The response rate also risks producing a non-representative sample, affecting the generalisability of the results. Furthermore, this study captures only the healthcare worker perspective, and future research should incorporate patient and societal viewpoints. Additional, sub-group or regression analysis to explore statistically significant differences and outliers is also warranted.

## Conclusion

The study highlights widespread overestimation of CPR effectiveness, and a significant misunderstanding of the restrictiveness of DNACPR decisions among healthcare professionals. Effective communication and comprehensive training are crucial to overcome communication challenges, which remains a key barrier. While national policies provide important guidance, greater awareness and understanding of frameworks is necessary.

## Availability of data and materials

Data are available on request from the corresponding author.

## Funding information

This work was funded by the HSE National Office for Human Rights and Equality Policy.

## CRediT authorship contribution statement

**John Lombard:** Writing – review & editing, Writing – original draft, Methodology, Investigation, Funding acquisition, Formal analysis, Conceptualization. **Hope Davidson:** Writing – review & editing, Writing – original draft, Methodology, Investigation, Formal analysis. **Owen Doody:** Writing – review & editing, Methodology, Investigation, Formal analysis.

## Declaration of competing interest

The authors declare the following financial interests/personal relationships which may be considered as potential competing interests: John Lombard reports financial support was provided by Health Service Executive. If there are other authors, they declare that they have no known competing financial interests or personal relationships that could have appeared to influence the work reported in this paper.
