# Supplementary material for: A survey study of healthcare workers on do not Attempt cardiopulmonary resuscitation practice and policy in Ireland
Source: Resusc Plus. 2024 Oct 17;20:100799. doi: 10.1016/j.resplu.2024.100799 (PMC11513516; doi:10.1016/j.resplu.2024.100799)
Supplement: Supplementary Data 1 [file mmc1.docx]

Do Not Attempt Cardiopulmonary Resuscitation (DNACPR) Practice and Policy (Service Provider)

Start of Block: Welcome page

**Do Not Attempt Cardiopulmonary Resuscitation (DNACPR) Practice and Policy**

Welcome to this questionnaire on the topic of Do Not Attempt Cardiopulmonary Resuscitation (DNACPR) practice and policy.

The research aims to capture the perspective of healthcare workers on current DNACPR practice and policy in Ireland.

Participation in the questionnaire is voluntary and anonymous. You have the right not to answer any of the questions and can withdraw from the questionnaire at any point prior to submission.

The questionnaire will take 10-15 minutes to complete.

The information gathered will be included in a report for the Health Service Executive and will inform subsequent policy development. The information may also be published as part of academic publications and/or presentations. Nothing in the publications or presentations will be used to identify you.  

This research study has received ethics approval from the HSE Mid-Western Area Research Ethics Committee (ref: 055/2023).

If you have any queries or would like more information about the project before you agree to participate, please contact:

I declare that I have been fully briefed on the nature of this study and my role in it. I have full knowledge of how the information will be used and that it will be stored confidentially. I am aware that this information may be used in future academic presentations and publications about this study. I fully understand that I am free to withdraw my participation without having to explain or give a reason.

- Yes (1)
- No (2)

Skip To: End of Survey If I declare that I have been fully briefed on the nature of this study and my role in it. I have fu... = No

End of Block: Welcome page

Start of Block: Demographic and Career Characteristics

Q1 Gender:

- Man (1)
- Woman (2)
- Non-binary (3)
- Prefer not to say (4)
- I identify my gender as: (5) __________________________________________________

Q2 Age:

- 18-29 years (1)
- 30-39 years (2)
- 40-49 years (3)
- 50-59 years (4)
- 60-69 years (5)
- 70-79 years (6)
- 80+ years (7)

Q3 Profession:

- Doctor (1)
- Nurse (2)
- Paramedic (3)
- Other, please specify: (4) __________________________________________________

Q4 Job title:

- Intern (1)
- SHO (2)
- Registrar (3)
- SpR (4)
- Consultant (5)
- General Practitioner (6)
- Staff nurse (7)
- CNM (8)
- CNS (9)
- ANP (10)
- EMT (11)
- Paramedic (12)
- Advanced Paramedic (13)
- Other, please specify: (14) __________________________________________________
- Not applicable (15)

Q5 Specialty / Division (*if applicable*):

________________________________________________________________

Q6 Years of practice:

- Less than one year (1)
- 1-5 years (2)
- 6-10 years (3)
- 11-15 years (4)
- 16+ years (5)

Q7 Where do you currently practice: [*Check all that apply*]

- Hospital (1)
- Nursing home (2)
- GP practice (3)
- Ambulance service (4)
- Community setting (5)
- Other, please specify (6) __________________________________________________

Q8 Country where you obtained your first medical qualification:

- Ireland (1)
- United Kingdom (2)
- United States (3)
- Australia (4)
- India (5)
- Philippines (6)
- Pakistan (7)
- Sudan (8)
- Other, please specify (9) __________________________________________________

Q9 In the past 12 months, have you been involved in a Do Not Attempt Cardiopulmonary Resuscitation (DNACPR) decision-making process as a healthcare worker?

- Yes (1)
- No (2)
- Not sure (3)

End of Block: Demographic and Career Characteristics

Start of Block: Do Not Attempt Cardiopulmonary Resuscitation (DNACPR) Knowledge and Policy

Q10 How familiar are you with the following:

|  | Not at all familiar (1) | Slightly familiar (2) | Moderately familiar (3) | Very familiar (4) | Extremely familiar (5) |
| --- | --- | --- | --- | --- | --- |
| HSE National Consent Policy 2022 (1) |  |  |  |  |  |
| Guidance on 'Do Not Attempt Resuscitation' contained in the HSE National Consent Policy 2022 (2) |  |  |  |  |  |
| HSE Guidance Regarding Cardiopulmonary Resuscitation and DNAR Decision-Making during the COVID-19 Pandemic (3) |  |  |  |  |  |
| The Assisted Decision-Making (Capacity) Act 2015 (4) |  |  |  |  |  |
| Advance healthcare directives (5) |  |  |  |  |  |
| Decision support arrangements under the Assisted Decision-Making (Capacity) Act (e.g. decision-making assistant; co-decision-maker; decision-making representative; designated healthcare representative) (6) |  |  |  |  |  |

Q11 How would you describe your knowledge of DNACPR decision-making?

- Very poor (1)
- Poor (2)
- Fair (3)
- Good (4)
- Excellent (5)

Q12 Which in your opinion is a more accurate understanding of a DNACPR decision:

- No chest compressions, defibrillation or artificial ventilation in the event of cardiopulmonary arrest (1)
- Limitation of measures to preserve life which extend beyond resuscitation (2)
- No effort should be made to preserve life (3)

Q13 What is your best estimate as to the rate of survival to hospital discharge for a person who receives CPR during an in-hospital cardiac arrest?

- Less than 15% (1)
- 15-34% (2)
- 35-49% (3)
- 50%+ (4)

Q14 What is your best estimate as to the rate of survival through to hospital admission and discharge for a person who receives CPR during an out-of-hospital cardiac arrest?

- Less than 10% (1)
- 11-29% (2)
- 30-49% (3)
- 50%+ (4)

Q15 What affect, if any, do you think a DNACPR decision would have on the quality of a patient's care:

- Positive affect (1)
- Negative affect (2)
- No affect (3)

Q16 If a patient has a DNACPR decision, can they be referred to hospital from home / nursing home?

- Yes (1)
- No (2)
- Not sure (3)

Q17 How important do you think the following elements are in informing DNACPR decision-making?

|  | Not at all important (1) | Slightly important (2) | Moderately important (3) | Very important (4) | Extremely important (5) |
| --- | --- | --- | --- | --- | --- |
| National policy, e.g. HSE National Consent Policy 2022 (1) |  |  |  |  |  |
| Local policy (2) |  |  |  |  |  |
| Patient wishes and preferences (3) |  |  |  |  |  |
| Advance healthcare directive (4) |  |  |  |  |  |
| Patient's quality of life (5) |  |  |  |  |  |
| Input of patient's family members (6) |  |  |  |  |  |
| Input of decision supporters under the Assisted Decision-Making (Capacity) Act (e.g. decision-making assistant; co-decision-maker; decision-making representative; designated healthcare representative) (7) |  |  |  |  |  |
| Professional standards / code of conduct (8) |  |  |  |  |  |
| Personal clinical judgement (9) |  |  |  |  |  |
| Input from other healthcare workers (10) |  |  |  |  |  |

End of Block: Do Not Attempt Cardiopulmonary Resuscitation (DNACPR) Knowledge and Policy

Start of Block: Timing of Do Not Attempt Cardiopulmonary Resuscitation (DNACPR) Decision-Making

Q18 When is the most appropriate time to start a discussion about, or introduce the concept of DNACPR? [*Check all that apply*]

- While attending the GP surgery (1)
- After a person is diagnosed with a terminal illness (2)
- During an outpatient clinic appointment (3)
- After a person is admitted to hospital (4)
- At the time of consent for surgery (5)
- Other, please specify (6) __________________________________________________
- Not sure (7)

Q19 In total, how much time do you usually spend discussing resuscitation status with a patient?

- less than one minute (1)
- 1-5 mins (2)
- 6-10 mins (3)
- 11-15 mins (4)
- 15+ mins (5)
- Not applicable (6)

Q20 In the following situations, how likely is it that a documented DNACPR decision would be reassessed?

|  | Extremely unlikely (1) | Somewhat unlikely (2) | Neither likely nor unlikely (3) | Somewhat likely (4) | Extremely likely (5) |
| --- | --- | --- | --- | --- | --- |
| The patient's clinical condition deteriorates (1) |  |  |  |  |  |
| The patient's clinical condition improves (2) |  |  |  |  |  |
| The patient's preferences regarding CPR change (3) |  |  |  |  |  |
| A patient has recovered capacity in respect of a DNACPR decision (4) |  |  |  |  |  |
| An advance healthcare directive is identified (5) |  |  |  |  |  |
| Clinical responsibility for the patient changes (6) |  |  |  |  |  |
| The patient is to undergo a medical or surgical procedure (7) |  |  |  |  |  |
| The patient has a prolonged stay in hospital or is a nursing home resident (8) |  |  |  |  |  |
| At time of hospital discharge (9) |  |  |  |  |  |

Q21 Would a documented DNACPR decision from a prior hospital admission be applied if the patient is readmitted to the hospital?

- Yes (1)
- No (2)
- Not sure (3)

End of Block: Timing of Do Not Attempt Cardiopulmonary Resuscitation (DNACPR) Decision-Making

Start of Block: Involvement in DNACPR decision-making

Q22 In circumstances where the patient has decision-making capacity, how often is the patient involved in the DNACPR decision-making process?

- Never (1)
- Rarely (2)
- Sometimes (3)
- Often (4)
- Always (5)
- Not sure (6)

Q23 How often is the patient offered the opportunity to have a family member, friend, or decision supporter involved in the DNACPR decision-making process?

- Never (1)
- Rarely (2)
- Sometimes (3)
- Often (4)
- Always (5)
- Not sure (6)

Q24 Where CPR is judged inappropriate, how often is this decision communicated to the patient?

- Never (1)
- Rarely (2)
- Sometimes (3)
- Often (4)
- Always (5)
- Not sure (6)

Q25 A patient’s refusal to participate in the DNACPR decision-making process should be respected:

- Strongly disagree (1)
- Somewhat disagree (2)
- Neither agree nor disagree (3)
- Somewhat agree (4)
- Strongly agree (5)
- Not sure (6)

Q26 A patient does not need to be included in the DNACPR decision-making process if this would be upsetting for them:

- Strongly disagree (1)
- Somewhat disagree (2)
- Neither agree nor disagree (3)
- Somewhat agree (4)
- Strongly agree (5)
- Not sure (6)

Q27 If a DNACPR decision relates to a child or young person, how important is it to facilitate their involvement in the decision-making process?

|  | Not at all important (1) | Slightly important (2) | Moderately important (3) | Very important (4) | Extremely important (5) | Not sure (6) |
| --- | --- | --- | --- | --- | --- | --- |
| Child is 0-5 years (1) |  |  |  |  |  |  |
| Child is 6-11 years (2) |  |  |  |  |  |  |
| Child is 12-15 years (3) |  |  |  |  |  |  |

Q28 If a DNACPR decision relates to a child or young person, how important is it to facilitate the involvement of their parents/guardians in the decision-making process?

|  | Not at all important (1) | Slightly important (2) | Moderately important (3) | Very important (4) | Extremely important (5) | Not sure (6) |
| --- | --- | --- | --- | --- | --- | --- |
| Child is 0-5 years (1) |  |  |  |  |  |  |
| Child is 6-11 years (2) |  |  |  |  |  |  |
| Child is 12-15 years (3) |  |  |  |  |  |  |

Q29 Have you experienced tensions with the patient, family members, or decision supporters in respect of a DNACPR decision?

- Yes (1)
- No (2)
- Not sure (3)

Skip To: Q35 If Have you experienced tensions with the patient, family members, or decision supporters in respect... = Yes

Skip To: End of Block If Have you experienced tensions with the patient, family members, or decision supporters in respect... = No

Skip To: End of Block If Have you experienced tensions with the patient, family members, or decision supporters in respect... = Not sure

Q30 Can you please outline the nature and cause of these tensions?

________________________________________________________________

End of Block: Involvement in DNACPR decision-making

Start of Block: Do Not Attempt Cardiopulmonary Resuscitation (DNACPR) Discussion

Q31 How comfortable are you discussing DNACPR with the patient?

- Very uncomfortable (1)
- Somewhat uncomfortable (2)
- Neither comfortable nor uncomfortable (3)
- Somewhat comfortable (4)
- Very comfortable (5)
- Not applicable (6)

Q32 How would you rate your communication skills for a DNACPR discussion with a patient?

- Very poor (1)
- Poor (2)
- Fair (3)
- Good (4)
- Excellent (5)
- Not sure (6)

Q33 When discussing DNACPR, do you enquire as to whether the patient has an advance healthcare directive?

- Never (1)
- Rarely (2)
- Sometimes (3)
- Often (4)
- Always (5)
- Not applicable (6)

Q34 Who should initiate the DNACPR discussion? [Check all that apply]

- The patient (1)
- Senior healthcare professional (2)
- General practitioner (3)
- Nurse (4)
- Other, please specify (5) __________________________________________________
- Not sure (6)

End of Block: Do Not Attempt Cardiopulmonary Resuscitation (DNACPR) Discussion

Start of Block: Communication of and adherence to the DNACPR decision

Q35 The DNACPR decision is always communicated to the relevant healthcare workers:

- Yes (1)
- No (2)
- Not sure (3)

Skip To: Q43 If The DNACPR decision is always communicated to the relevant healthcare workers: = Not sure

Display This Question:

If The DNACPR decision is always communicated to the relevant healthcare workers: = Yes

Q36 What do you think facilitates this communication between healthcare workers in the context of DNACPR decisions?

________________________________________________________________

Display This Question:

If The DNACPR decision is always communicated to the relevant healthcare workers: = No

Q37 What do you consider to be the barriers to effective communication between healthcare workers in the context of DNACPR decisions?

________________________________________________________________

Q38 If there is a disagreement about the balance of benefits and risks of CPR, an offer of a second, independent opinion should be made to the patient:

- Yes (1)
- No (2)
- Not sure (3)

Q39 A person may experience a cardiorespiratory arrest from a readily reversible cause which is unconnected to their underlying illness/condition. In this situation would an existing DNACPR decision always be followed?

- Yes (1)
- No (2)
- Not sure (3)

End of Block: Communication of and adherence to the DNACPR decision

Start of Block: Closing

Q40 Is there anything you would like to add on the topic of Do Not Attempt Cardiopulmonary Resuscitation (DNACPR) practice and policy? If not, please click the forward arrow to record your questionnaire responses.

________________________________________________________________

End of Block: Closing
